# FACT and Ash1 promote long-range and bidirectional nucleosome eviction at the *HO* promoter

**DOI:** 10.1093/nar/gkaa819

**Published:** 2020-10-03

**Authors:** Yaxin Yu, Robert M Yarrington, David J Stillman

**Affiliations:** Department of Pathology, University of Utah Health Sciences Center, Salt Lake City, UT 84112, USA; Department of Pathology, University of Utah Health Sciences Center, Salt Lake City, UT 84112, USA; Department of Pathology, University of Utah Health Sciences Center, Salt Lake City, UT 84112, USA

## Abstract

The *Saccharomyces cerevisiae HO* gene is a model regulatory system with complex transcriptional regulation. Budding yeast divide asymmetrically and *HO* is expressed only in mother cells where a nucleosome eviction cascade along the promoter during the cell cycle enables activation. *HO* expression in daughter cells is inhibited by high concentration of Ash1 in daughters. To understand how Ash1 represses transcription, we used a *myo4* mutation which boosts Ash1 accumulation in both mothers and daughters and show that Ash1 inhibits promoter recruitment of SWI/SNF and Gcn5. We show Ash1 is also required for the efficient nucleosome repopulation that occurs after eviction, and the strongest effects of Ash1 are seen when Ash1 has been degraded and at promoter locations distant from where Ash1 bound. Additionally, we defined a specific nucleosome/nucleosome-depleted region structure that restricts *HO* activation to one of two paralogous DNA-binding factors. We also show that nucleosome eviction occurs bidirectionally over a large distance. Significantly, eviction of the more distant nucleosomes is dependent upon the FACT histone chaperone, and FACT is recruited to these regions when eviction is beginning. These last observations, along with ChIP experiments involving the SBF factor, suggest a long-distance loop transiently forms at the *HO* promoter.

## INTRODUCTION

Eukaryotic transcription is regulated in complex ways, using coactivators and corepressors to control recruitment of the transcriptional machinery to a chromatin template (1,2). The budding yeast Saccharomyces cerevisiae has long been used as a system to study eukaryotic transcriptional regulation (3). While most yeast genes have small promoters, the extensively studied *HO* gene has complex regulation and a regulatory region 5–10 times larger than the average promoter (4).

*HO* encodes the endonuclease that cleaves DNA at the mating type (*MAT*) locus and initiates mating type interconversion (5). Budding yeast divide asymmetrically, producing large mother and small daughter cells. One unique feature of *HO* regulation is that *HO* is only expressed in haploid mother cells, and thus only mother cells are capable of switching their mating type (6,7). *HO* is cell-cycle regulated and is expressed only in late G1 phase in mother cells. Transcriptional activation of *HO* requires multiple transcription factors and coactivators acting sequentially during the cell cycle (8–10). The initiating event occurs in M phase when the Swi5 DNA-binding protein enters the nucleus and binds at two sites within the more distal URS1 region of the promoter. Swi5 recruits three coactivator complexes to URS1, the SWI/SNF chromatin remodeler, the SAGA complex containing the Gcn5 histone acetyltransferase and Mediator (8,9). SWI/SNF and FACT facilitate a cascade of nucleosome evictions along the promoter, first at the URS1 region of the promoter, then at the left end of the more proximal URS2 region and finally at the right half of URS2 (10). This nucleosome eviction allows the SBF factor to bind to sites within URS2 and ultimately activate transcription (11,12).

The Swi5 transcriptional activator that initiates the program of *HO* gene activation is related to a paralogous yeast transcription factor, Ace2, as both proteins have identical DNA binding domains and recognize the same DNA sequences *in vitro* (13,14). Additionally, both proteins are cell cycle regulated in a similar fashion (15,16), although Ace2 accumulates primarily in daughter cells (17). It is not surprising that Swi5 and Ace2, with identical DNA binding domains, activate some of the same target genes; however, Swi5 and Ace2 each also have unique target genes (15). Ace2 activates a set of daughter specific genes (18); ChIP experiments show Swi5 binds to these daughter-specific genes *in vivo*, but fails to activate due to co-occupancy in these promoters by the Fkh inhibitory factor (15). Although Ace2 can bind to the *HO* promoter *in vitro*, Ace2 does not bind *in vivo* and fails to activate *HO* expression. The mechanism that prevents Ace2 from binding to the *HO* promoter *invivo* has been a mystery, up to now.

The *ASH1* repressor gene was identified by genetic screens for mutations that allow *HO* expression in daughter cells (19,20). *ASH1* encodes a DNA-binding protein that localizes primarily to daughter cells (19,20), binds to the *HO* promoter (21–23) and represses *HO* expression in daughters by recruiting the Rpd3 histone deacetylase (21,23) and the Tup1 repressor (manuscript in preparation). Ash1 is often described as a ‘daughter-specific’ repressor, but this term is not fully accurate as Ash1 is not localized exclusively to daughter cells. Quantitation of Ash1 localization by immunofluorescence microscopy shows that Ash1 is present in both mother and daughter cells, though substantially more protein is present in daughters (20). Ash1 also represses *HO* expression in mother cells, as an *ash1* mutation increases the frequency of mating type switching in mother cells (19,20), and an *ash1* mutation allows *HO* expression in mothers in the absence of the normally required Gcn5 acetyltransferase (24). Thus, Ash1 acts in both daughter and mother cells, but has a more significant role in daughters. The high concentration of Ash1 in daughter cells blocks *HO* expression, whereas in mother cells Ash1 merely contributes to making chromatin in the *HO* promoter repressive without precluding the possibility of expression.

The asymmetry in Ash1 protein localization results from the *ASH1* gene being transcribed in late M phase and the *ASH1* mRNA being transported to the bud tip in daughter cells where it is translated into protein (25,26). This mRNA transport results in a much higher concentration of Ash1 in daughters compared to mothers, effectively blocking *HO* expression in daughters (27). A genetic screen identified five *SHE* genes required for proper Ash1 localization, including *MYO4 (SHE1)*, an unconventional myosin (28). The She proteins bind to elements in the *ASH1* mRNA, and thus link the mRNA to the actin cables that function to transport vesicles to the growing bud tip in daughter cells (29).

FACT is a conserved histone chaperone composed of Spt16 and Pob3 subunits (30). FACT contains multiple histone binding modules, and it can both destabilize and assemble nucleosomes (31–34). We have previously shown that FACT is required for *HO* expression (35) and that the complete eviction of nucleosomes that occurs as a prelude to *HO* promoter activation is dependent upon FACT (10).

In this paper we first address the mechanisms by which Ash1 represses transcription using a *myo4* mutation that increases Ash1 concentration in mother cells. We show Ash1 inhibits recruitment of SWI/SNF and Gcn5 to the *HO* promoter, and that Ash1 is required for efficient repopulation of nucleosomes following eviction. Importantly, the strongest effects of Ash1 are seen at promoter locations distant from where Ash1 binds and at a time when Ash1 is no longer present in the cell. We also demonstrate that nucleosomes are bidirectionally evicted over a large distance at the *HO* promoter, and that FACT is required for effective eviction of the nucleosomes distant from the site where chromatin remodelers are first recruited. Additionally, evidence is presented suggesting that a loop forms at the *HO* promoter, and the bidirectional eviction of nucleosomes could promote formation of this loop. Finally, we identify an unusual chromatin structure at the *HO* promoter that is required to prevent Ace2 from binding and activating *HO* transcription.

## MATERIALS AND METHODS

All yeast strains used in this study are listed in Supplementary Table S1 and are isogenic in the W303 background (36). Standard genetic methods were used for strain construction (37,38). The *ASH1-V5*, *GCN5-V5*, *SWI2-V5* and *SWI5-Myc* C-terminal epitope tags have been described previously (10,21,39). The *SPT16-Myc* C-terminal epitope tag were added as described (40) using plasmid pFA6a:13Myc:KanMX6 (41). The *HO(10XSBFmut)::(3′)KanMX* allele with mutations at ten SBF binding sites was created from the *HO(9XSBFmut)* promoter mutant (21) by the *delitto perfetto* method (42), mutating the possible SBF site at −1166 (23). The *HO-CLN2* hybrid promoter (*HO[-1725 to −1398 deleted]:CLN2[−764 to −435, mutSBF-wtNDR]:: KanMX(3′))* was also created by the *delitto perfetto* method, replacing nt −1725 to −1398 of *HO* with nt −764 to −435 of a version of the *CLN2* promoter with mutations in the three SBF binding sites (43). The *HO::KanMX(3′), HO(10XSBFmut)::(3′)KanMX* and *HO[−1725 to −1398 deleted]:CLN2[−764 to −35, mutSBF-wtNDR]:: KanMX(3′)* alleles have a KanMX marker inserted 3*′* to the *HO* ORF, so that the allele can be followed in crosses (21).

Cell-cycle synchronization was performed by galactose withdrawal and re-addition using a *GALp::CDC20* strain grown at 25°C in YP medium containing 2% galactose and 2% raffinose (9). Cell-cycle synchrony was confirmed by examination of budding indices and analysis of cycle-regulated mRNAs. In all other experiments, cells were grown at 30°C in YPAD medium (38).

ChIPs were performed as described (9,15) using a mouse monoclonal antibody to the V5 epitope (SV5-Pk1, Abcam), the Myc epitope (4A6; Upstate), or anti-histone H3 (07–690, Upstate) and Rabbit and Pan Mouse IgG-coated magnetic beads (Life Technologies). Samples prepared for ChIPs were cross-linked in 1% formaldehyde overnight on ice. ChIP assays were analyzed by real time quantitative polymerase chain reaction (qPCR) as described (44). As indicated in figure legends, the ChIP samples were first normalized either to the ChIP signal for a negative control region, such as at the IGR-I gene-free reference region on chromosome I (45), or a positive control region; in both cases the ChIP values were also normalized to their respective input DNA sample. Error bars reflect the standard deviation of at least three biological samples. *P*-values were calculated by paired *t*-tests.

RNA was isolated from either synchronized or logarithmically growing cells, and *HO* mRNA levels were measured by RT-qPCR as described (15). For all logarithmically grown strains, RNA expression was normalized to *RPR1* expression and graphed relative to wild-type. For the synchrony experiment, RNA expression was normalized to *RPR1* expression and graphed relative to the 0 min WT expression. Error bars reflect the standard deviation of at least three biological samples. *P*-values were calculated by paired *t*-tests.

MNase mapping of nucleosome positions was performed as described previously (46). DNA was extracted from mononucleosomes prepared using a modified ChIP protocol (9,15) in which cells were formaldehyde cross-linked for only 5 min at room temperature and chromatin was only lightly sheared prior to micrococcal nuclease digestion. Anti-histone H3 (07–690; Upstate) and antibody-coated magnetic beads (rabbit IgG beads; Life Technologies) were used for ChIP, and isolated mononucleosomes were analyzed by real-time qPCR as described previously (44).

Oligonucleotides used for ChIP, RT-qPCR and MNase mapping are listed in Supplementary Table S2.

## RESULTS AND DISCUSSION

### A *myo4* mutation reduces *HO* expression

**Figure 1.**
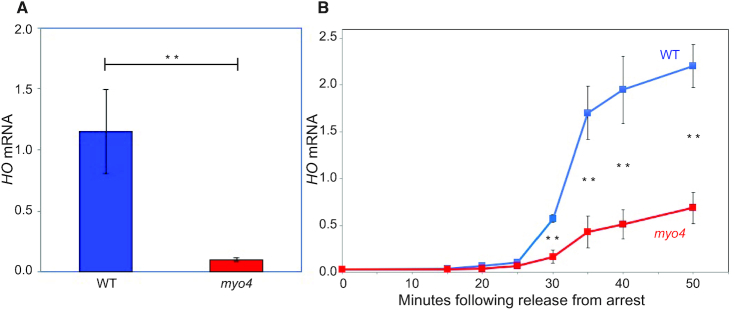
*HO* Expression is reduced in a *myo4* mutant. (**A**) *HO* mRNA levels were measured from log phase wild-type and *myo4* mutant cells by RT-qPCR. The error bars reflect the standard deviation of three biological samples. **P < 0.01. (**B**) *HO* mRNA levels were measured for wild-type and *myo4* mutant cells synchronized with a *GALp:CDC20* arrest and release. The error bars reflect the standard deviation of three biological samples. **P < 0.01.

The *MYO4* gene was identified through mutations that reduce mating type switching in mother cells (28) and also affect the asymmetric localization of Ash1 protein and mRNA (19,25,47–48). We used RT-qPCR to determine whether a *myo4* mutation affects *HO* expression. As shown in Figure 1A, *HO* expression is reduced by 90% in a *myo4* mutant strain. We also examined *HO* expression during the cell cycle, using cells synchronized with a *GALp::CDC20* arrest and release protocol (Figure 1B). This experiment also shows a major decrement in *HO* expression in a *myo4* mutant.

**Figure 2.**
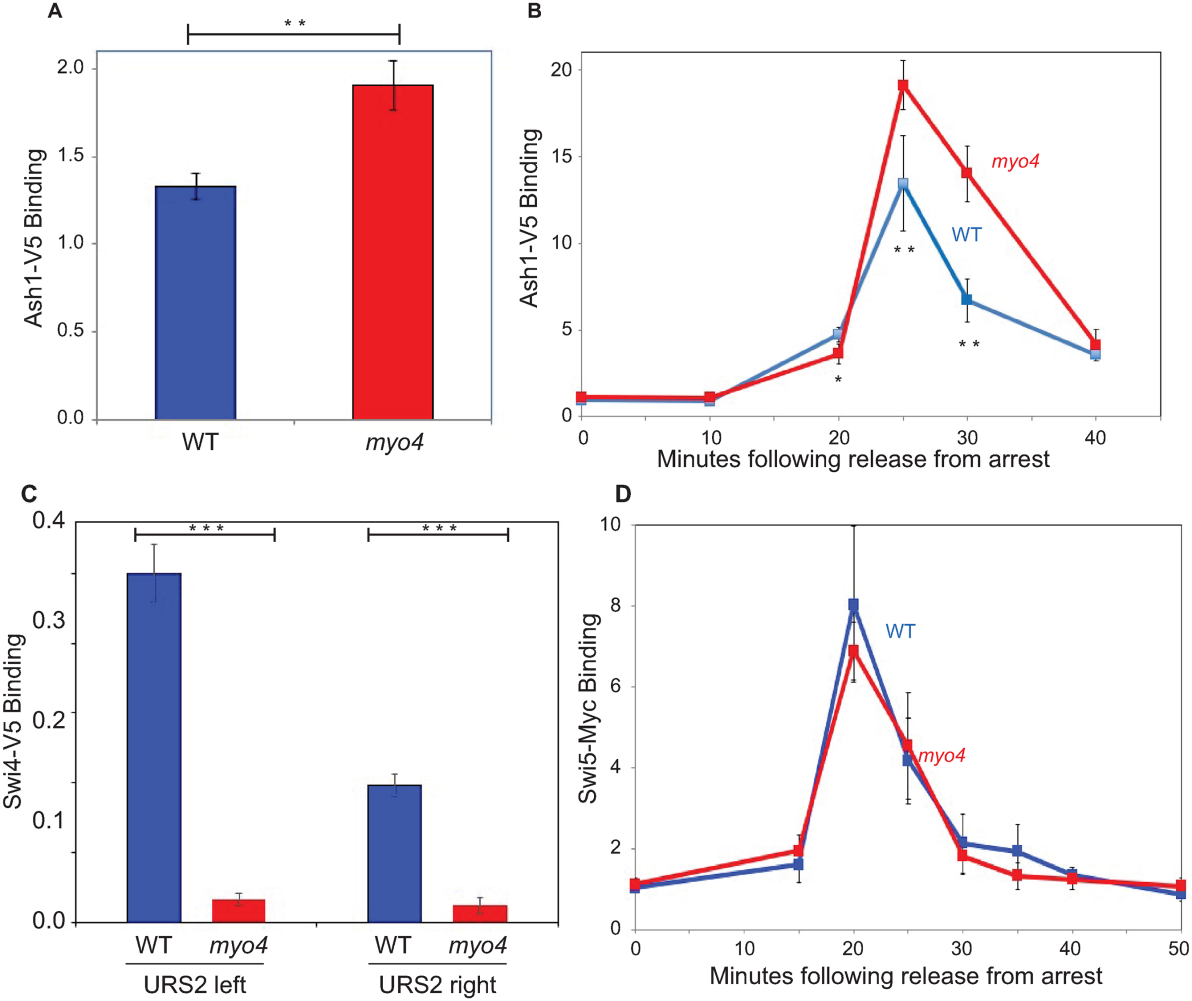
A *myo4* mutation has opposite effects on Ash1 and SBF binding to the *HO* promoter. (**A**) Ash1 binding to the *HO* promoter was measured by ChIP assays of Ash1-V5 in log phase wild-type and *myo4* mutant log phase cells. ChIP values were first normalized to the ChIP signal at the *CLN3* positive control region. The error bars reflect the standard deviation of three biological samples. **P < 0.01. (**B**) Ash1 binding to the *HO* promoter was measured by ChIP assays of Ash1-V5 in wild-type and *myo4* mutant cells synchronized with a *GALp:CDC20* arrest and release. ChIP values were first normalized to the ChIP signal at the *ACT1* negative control region. The error bars reflect the standard deviation of three biological samples. *P < 0.05, **P < 0.01. (**C**) A *myo4* mutation results in decreased SBF binding. SBF binding to the URS2-left and URS2-right regions of the *HO* promoter was measured by ChIP assays of the Swi4-V5 subunit of SBF in log phase wild-type and *myo4* mutant cells. ChIP values were first normalized to the ChIP signal at the *CLN2* positive control region. The error bars reflect the standard deviation of three biological samples. ***P < 0.001. (**D**) A *myo4* mutation does not affect Swi5 binding. Swi5-Myc binding to the URS1 region of the *HO* promoter was measured by ChIP assays in wild-type and *myo4* mutant cells synchronized with a *GALp:CDC20* arrest and release. ChIP values were first normalized to the ChIP signal at the IGR-I negative control region. The error bars reflect the standard deviation of three biological samples.

Previous studies have shown that a *myo4* deletion mutation increases the amount of Ash1 protein present in mother cells (19), and we tested whether the loss of Myo4 causes an increase in the amount of Ash1 bound to the *HO* promoter. A ChIP assay shows a modest but significant increase in Ash1-V5 binding to *HO* in the *myo4* mutant (Figure 2A). An experiment with synchronized cells shows that Ash1-V5 binding peaks at the same time in wild-type and *myo4* cells, but that Ash1-V5 appears to persist longer in the cell cycle in the *myo4* mutant (Figure 2B). Ash1 is abundant in daughter cells in wild-type, so it is likely that increased Ash1 in mother cells is responsible for the decreased *HO* expression in a *myo4* mutant.

### A *myo4* mutation decreases SBF binding to the *HO* promoter

Why does increased Ash1 at the promoter reduce *HO* expression? Since the SBF transcription factor, composed of the Swi4 and Swi6 subunits, is the proximal activator of *HO* (11,12), we performed ChIP experiments with a strain with Swi4-V5 to determine how a *myo4* mutation affects SBF binding to *HO* (Figure 2C). SBF binds more strongly to the left part of URS2 than to the right side, as has been observed previously (11,12). More importantly, the *myo4* mutation largely eliminates SBF binding at both parts of URS2, explaining why the *myo4* mutation blocks *HO* expression.

### A *myo4* mutation decreases SWI/SNF and SAGA binding to the *HO* promoter

**Figure 3.**
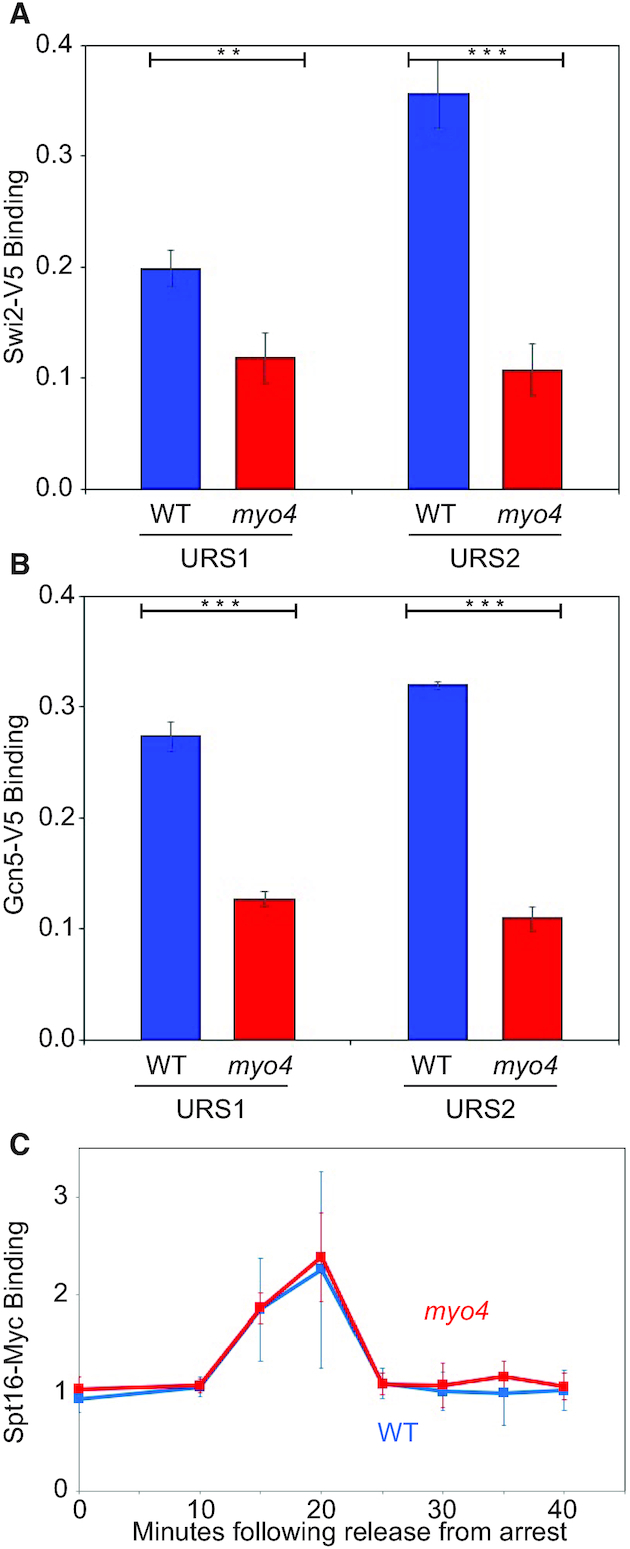
A *myo4* mutation results in decreased SWI/SNF and SAGA binding but does not affect FACT recruitment to the *HO* promoter. (**A**) A *myo4* mutation results in decreased SWI/SNF binding. SWI/SNF binding to the URS1 and URS2 regions of the *HO* promoter was measured by ChIP assays of the Swi2-V5 subunit of SWI/SNF in log phase wild-type and *myo4* mutant cells. ChIP values were first normalized to the ChIP signal at the *CLN2* positive control region. The error bars reflect the standard deviation of three biological samples. **P < 0.01, ***P < 0.001. (**B**) A *myo4* mutation results in decreased SAGA binding. Gcn5 binding to the URS1 and URS2 regions of the *HO* promoter was measured by ChIP assays of Gcn5-V5 in log phase wild-type and *myo4* mutant cells. ChIP values were first normalized to the ChIP signal at the *CLN2* positive control region. The error bars reflect the standard deviation of three biological samples. ***P < 0.001. (**C**) A *myo4* mutation does not affect FACT recruitment. Spt16-Myc binding to the URS2 region of the *HO* promoter was measured by ChIP assays in wild-type and *myo4* mutant cells synchronized with a *GALp:CDC20* arrest and release. ChIP values were first normalized to the ChIP signal at the IGR-I negative control region. The error bars reflect the standard deviation of three biological samples.

The next question concerns why SBF binding is reduced when there is additional Ash1. Swi5 binding at URS1 is the first event in *HO* activation (4). We therefore synchronized cells to compare Swi5-V5 binding at URS1 in wild-type and *myo4* strains (Figure 2D). The results show that Swi5 binding is unaffected by *myo4*. Swi5 recruits the SWI/SNF and SAGA coactivator complexes first to URS1 and then to URS2. We monitored binding with Swi2-V5 and Gcn5-V5 tags for the SWI/SNF and SAGA complexes, respectively (Figure 3A and B). The *myo4* mutation reduces, but does not eliminate, SWI/SNF and SAGA binding to both URS1 and URS2.

Because it is not clear whether the diminished recruitment of SWI/SNF and SAGA by a *myo4* mutation is sufficient to explain the complete elimination of SBF binding seen in Figure 2C, we examined other factors required for *HO* activation. FACT is transiently recruited to the *HO* promoter, and is required for the nucleosome eviction at the left end of URS2 that allows SBF to bind (10–12). To test whether a *myo4* mutation affected FACT recruitment, cells with an Spt16-Myc tag were arrested at G2/M and released to progress synchronously through the cell cycle. Samples were collected at time points after release, and FACT binding to URS2 peaks at 20 min after release (Figure 3C), as observed previously (10). Importantly, a *myo4* mutation has no effect on FACT recruitment. This is consistent with FACT being required for nucleosome eviction and *HO* activation rather than for nucleosome repopulation and *HO* repression.

### An *ash1* mutation affects nucleosome structure

**Figure 4.**
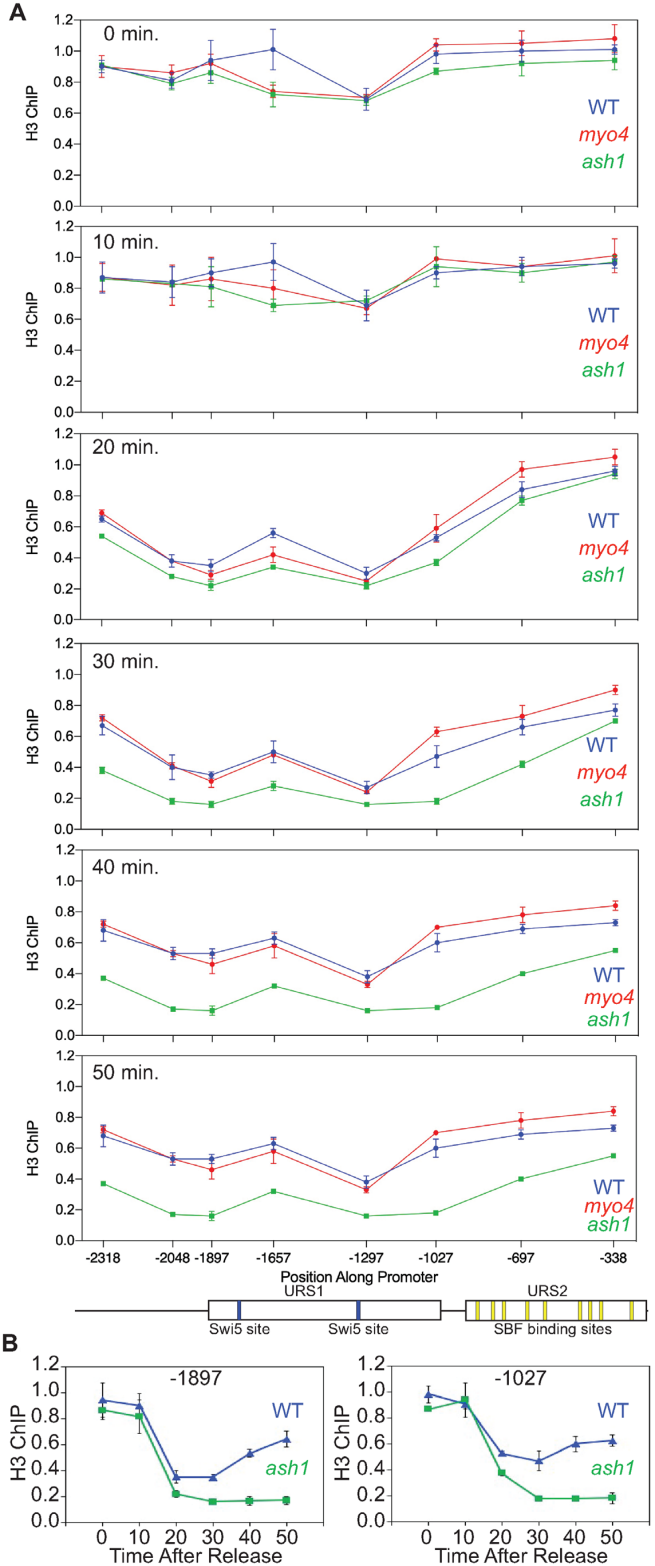
An *ash1* mutation affects nucleosome structure at *HO*, but a *myo4* mutation does not. (**A**) wild-type, *myo4* and *ash1* cells were synchronized with a *GALp:CDC20* arrest and release, and samples harvested at the indicated timepoints were used for H3 ChIP to determine nucleosome occupancy. PCR primers were used that span the *HO* promoter, with a promoter diagram at the bottom. wild-type blue, *myo4* red, *ash1* green. ChIP values were first normalized to the ChIP signal at the IGR-I negative control region. (**B**) The wild-type and *ash1* H3 ChIP data from for the −1897 and −1027 promoter positions are replotted as a function of time after release from the arrest. wild-type blue, *ash1* green.

*HO* promoter activation requires a cascade of nucleosome evictions along the promoter during the cell cycle, first at the URS1 region of the promoter, then at the left end of the more proximal URS2 region, and finally at the right half of URS2 (10). We asked whether excess Ash1 in mother cells, caused by a *myo4* mutation, or the absence of Ash1, in an *ash1* mutant, affected nucleosome eviction during the cell cycle. Cells were arrested at G2/M and then released, and samples of synchronized cells were used for histone H3 ChIP performed to measure histone occupancy at different time points (Figure 4A, also see Supplementary Figure S1). We expected a *myo4* mutation to reduce nucleosome eviction, as it causes a marked increase in Ash1 in mother cells; to our surprise there was little difference in nucleosome eviction between *myo4* and wild-type. In contrast, the *ash1* mutation has marked effects on nucleosome occupancy, particularly at 30 min (corresponding to G1 phase) and later time points. In wild-type cells, nucleosomes that had been evicted from the promoter earlier are repopulated to the promoter by the 40 and 50 min timepoints, but this does not occur in the *ash1* mutant (i.e. see promoter positions −1897 and −1027, Figure 4B). This indicates that Ash1 plays a role in this nucleosome repopulation, which is remarkable because Ash1 is not present at the *HO* promoter at these times; Ash1 is unstable (49) and ChIP experiments show that Ash1 binds only from 20–30 min after release from G2/M arrest (21), as shown in Supplementary Figure S2. Thus Ash1 has a prolonged effect on the *HO* promoter, with the effect on nucleosome occupancy persisting long after the Ash1 protein has been degraded.

The pattern of nucleosome occupancy at the *HO* promoter is therefore affected by the Ash1 protein. Ash1 is a sequence-specific DNA-binding transcriptional repressor that recruits the Rpd3 histone deacetylase (21,23) and the Tup1 repressor (manuscript in preparation). Using a *myo4* mutant that increases Ash1 concentration in mother cells, we show that Ash1 inhibits recruitment of SWI/SNF and Gcn5 to the *HO* promoter (Figure 3A and B) and that Ash1 is required for efficient repopulation of nucleosomes that occurs following eviction (Figure 4). We recently demonstrated that disruption of the *SIN4* subunit of Mediator also delays nucleosome repopulation at the *HO* promoter (39). Importantly, a *sin4* mutation results in increased recruitment of SWI/SNF and Gcn5 to the *HO* promoter (39) and an *ash1* mutation similarly increases SWI/SNF and Gcn5 recruitment (24). This suggests that an increase in the amount of SWI/SNF and Gcn5 delays nucleosome repopulation. Remarkably, the greatest effects of the *ash1* mutation are seen at a time in the cell cycle when Ash1 has been degraded and is no longer present in the cell. Additionally, Ash1 binds in the vicinity of −1890 and −1215 (manuscript in preparation), and thus the consequences of the *ash1* mutation are seen at promoter locations distant from where Ash1 binds. Thus, Ash1 can have long distance effects. Ash1 apparently causes hysteresis on the promoter, possibly through recruiting the Rpd3 histone deacetylase (23) that alters chromatin modification state in a durable way.

### Nucleosome-depleted regions (NDRs) surround a stable two nucleosome ‘Mesa’ required for proper regulation

**Figure 5.**
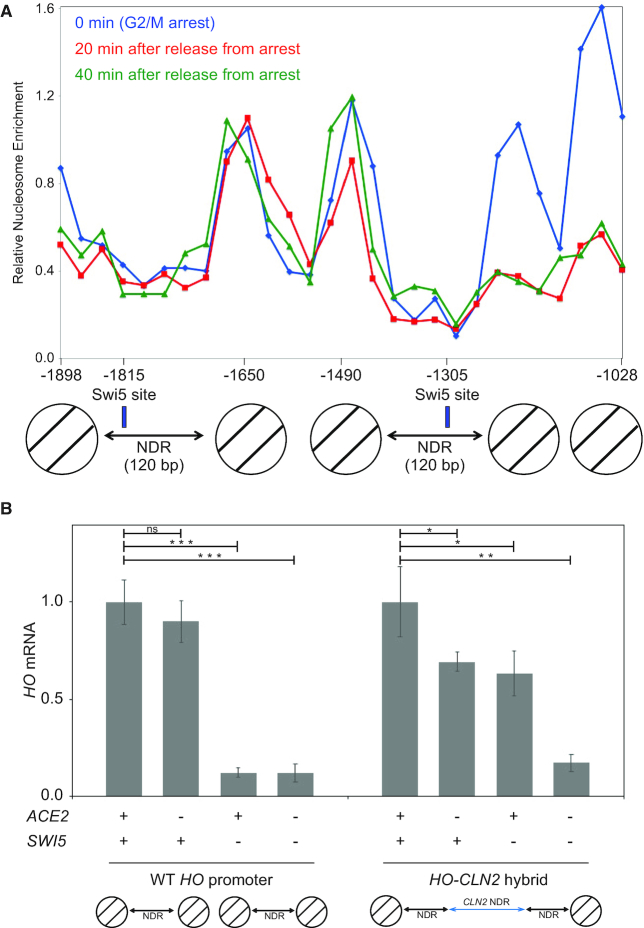
NDRs surround a stable two nucleosome ‘Mesa’ required for proper regulation. (**A**) MNase digestion experiment shows nucleosomes between the NDRs persist during the cell cycle. wild-type cells were synchronized with a *GALp:CDC20* arrest and release, and samples harvested at time points after release were treated with MNase and mononucleosomes isolated. Nucleosome positions along the *HO* promoter were determined using primers spaced every 30 bp. Blue, red and green lines indicate relative nucleosome enrichment as determined by H3 ChIP after 0, 20 and 40 min post arrest, respectively. Enrichment peaks indicate the midpoints of nucleosome positions, and determined nucleosome locations are represented below by circles with two diagonal lines, in agreement with previous work (12,57). Swi5 binding sites are indicated by blue rectangular boxes and determined NDRs are indicated by arrows. The distances of the Swi5 binding sites and nucleosomes from the *HO* ATG are indicated on the *X*-axis. (**B**) The two Mesa nucleosomes are required for promoter specific activation by Swi5 and not by Ace2. *HO* mRNA levels were measured by RT-qPCR from wild-type, *ace2*, *swi5* and *ace2 swi5* strains with either the native *HO* promoter or the *HO-CLN2* hybrid promoter lacking the two Mesa nucleosomes. Note that for each promoter, wild-type and the *HO-CLN2* hybrid, the data are normalized to the wild-type strain in each case. The error bars reflect the standard deviation of three biological samples. *P < 0.05, **P < 0.01, ***P < 0.001.

The effects of Ash1 in coordination with SWI/SNF, Gcn5 and Rpd3 suggest a primary role in modulating chromatin structure at the *HO* promoter, and this is consistent with the importance of FACT and Ash1 in removing and restoring nucleosome occupancy. To investigate this relationship further, we examined the importance of nucleosome positioning in this promoter. We have previously used MNase to map nucleosomes along the *HO* promoter, and determined that the Swi5 binding sites in URS1 are within nucleosome-depleted regions (NDRs) (12). Our previous H3 ChIP experiments suggested that the two nucleosomes between the two NDRs, centered at −1650 and −1490, respectively, are not evicted during the cell cycle (39). To address this question more directly, we performed a time-resolved MNase experiment. Cells were synchronized with *GALp::CDC20* arrest and release, and chromatin samples harvested at 0, 20 and 40 min after release were treated with MNase and mononucleosomal DNA purified. Nucleosome positions were determined using PCR with primer pairs every 30 bp along the promoter. Our nucleosome positioning results agree with genome-wide nucleosome occupancy studies (50,51). The results in Figure 5A show that the nucleosomes at −1650 and −1490 are not evicted, while those nucleosomes centered at −1208 and −1058 are evicted.

We refer to this unusual chromatin structure, with two stable nucleosomes flanked by two NDRs, as a ‘Mesa,’ since it rises above the surrounding chromatin landscape. To attempt to understand the role of these stable Mesa nucleosomes, we deleted the DNA corresponding to one stable nucleosome, but this substitution had no significant effect on *HO* expression (data not shown). We replaced the DNA sequence beneath these two nucleosomes with the sequence that positions two nucleosomes from the *CDC39* gene (12); this substitution had no effect on *HO* expression (data not shown), suggesting that the sequence itself may not be important for regulation but leaves the possibility that the presence of nucleosomes is important. We next eliminated the nucleosomes, using a 329 bp nucleosome free region from the *CLN2* promoter (43) to replace 329 bp containing the Mesa nucleosomes. This region of the *CLN2* promoter binds factors which confer the NDR feature, and this property of lacking nucleosomes is retained when this fragment is inserted to other genomic locations (11,43). The native promoter fragment contains three binding sites for the SBF activator, so to remove this complication we used a version lacking these SBF sites but still retains the desired property of low nucleosome occupancy (11,43).

Expression from this *HO-CLN2* hybrid promoter lacking the Mesa nucleosomes was substantially increased compared to the wild-type promoter (Supplementary Figure S3), suggesting that the Mesa nucleosomes have a role in limiting *HO* expression. The NDRs surrounding the Mesa nucleosomes have binding sites that have equal affinity for the paralogs Swi5 and Ace2 (13,14). Although Ace2 can bind to these *HO* promoter sites *in vitro*, Ace2 does not bind to these sites *in vivo*, and Ace2 is ineffective in activating *HO* transcription *in vivo* (13–15). One explanation for the increased expression from the *HO-CLN2* hybrid promoter is that the chromatin structure associated with the Mesa nucleosomes is the element that permits Swi5 binding but restricts Ace2.

To address this possibility, we constructed *ace2* and *swi5* mutant strains with the *HO-CLN2* hybrid promoter and compared expression to strains with the wild-type promoter (Figure 5B). As has been observed previously (13–15), expression of the wild-type *HO* promoter is unaffected by an *ace2* mutation but is eliminated in the *swi5* and *ace2 swi5* strains. In contrast, expression of the *HO-CLN2* hybrid promoter is modestly reduced in the *ace2* and *swi5* single mutants but sharply reduced in the *ace2 swi5* double mutant. Thus, while native *HO* can be activated by Swi5 but not by Ace2, the *HO-CLN2* hybrid promoter can be activated by either transcription factor. Thus the specific promoter structure of two nucleosomes flanked by NDRs with Swi5 bindings sites is essential for transcription factor specificity at the *HO* promoter.

### FACT-dependent bidirectional nucleosome eviction at the *HO* promoter

**Figure 6.**
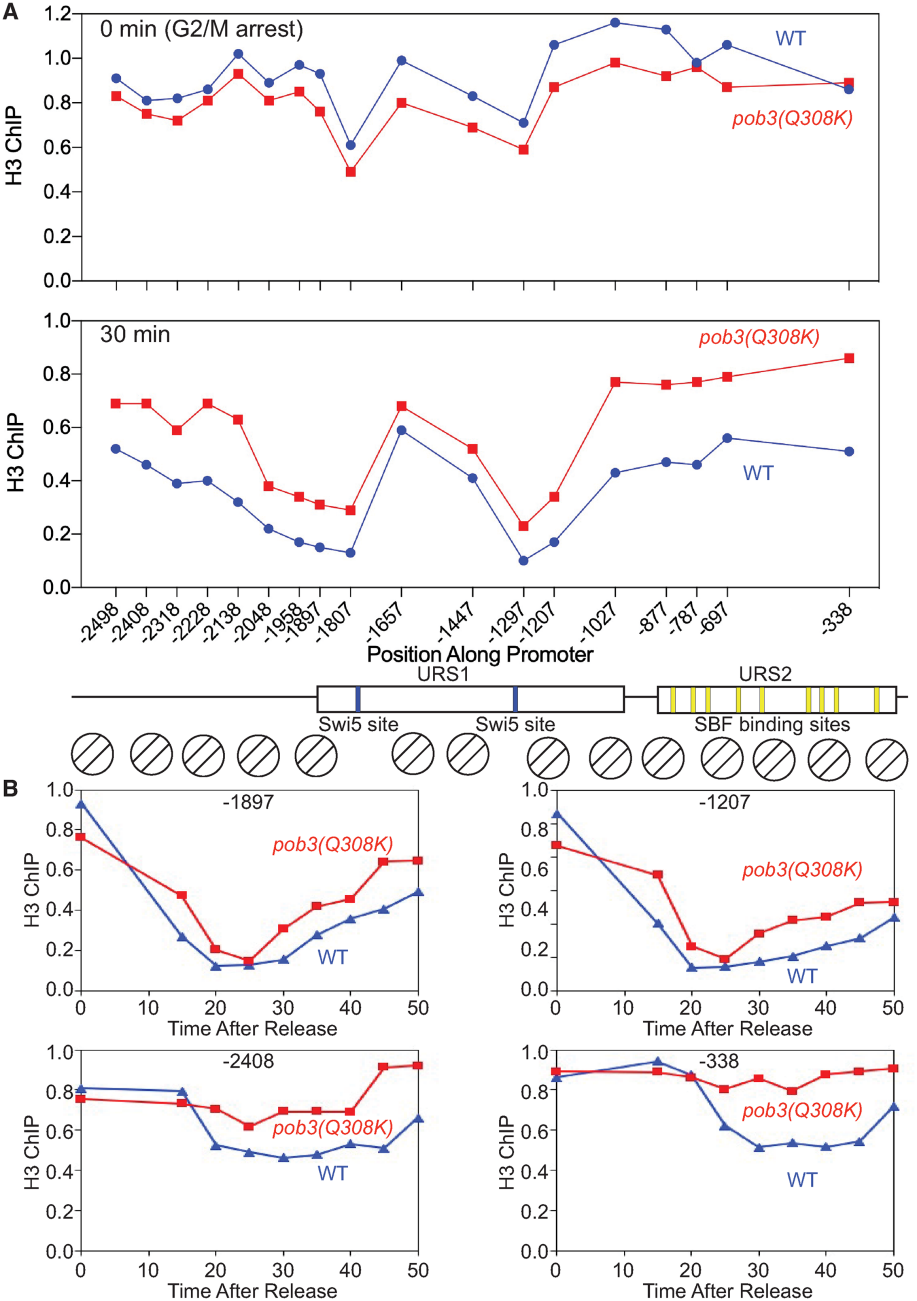
A *pob3-Q308K* mutation reduces nucleosome eviction in both directions. (**A**) wild-type and *pob3-Q308K* cells were synchronized with a *GALp:CDC20* arrest and release, and samples harvested at various timepoints were used for H3 ChIP to determine nucleosome occupancy. PCR primers were used that span the *HO* promoter, with a promoter diagram at the bottom. wild-type blue, *pob3-Q308K* red. ChIP values were first normalized to the ChIP signal at the IGR-I negative control region. Only the 0 min and 30 min time points are shown here; data for the other timepoints are shown in Supplementary Figure S2. The data replotted as a function of time are shown in Supplementary Figure S4. (**B**) The wild-type and *pob3-Q308K* H3 ChIP data for the −2408, −1897, −1027 and −338 promoter positions are replotted as a function of time after release from the arrest. wild-type blue, *pob3-Q308K* red.

In the H3 ChIP experiment (Figure 4A) we observed nucleosome eviction to the right of the Swi5 binding sites in URS1, toward the transcription start site, as seen previously (10). However, we also observed unexpected eviction to the left of these sites, distal to the gene. At positions −2318 and −2048, this eviction is more pronounced in an *ash1* mutant (Supplementary Figure S4). Our previous work only used primers sets from −1928 to −55 (10), and thus did not interrogate this promoter region to the left of URS1. We therefore performed a ChIP experiment using synchronized cells and primers extending to −2498. In addition to wild-type cells, we also tested a *pob3(Q308K)* FACT mutant, as our previous work showed that a *pob3(L78R)* mutation did not affect nucleosome eviction in URS1 but blocked eviction at the URS2 (10). The *pob3(Q308K)* FACT mutation is a better allele for experimental studies, as cells with the *pob3(L78R)* mutation grow slowly and the FACT complex is unstable, while *pob3(Q308K)* cells grow normally with wild-type levels of the FACT complex (52,53). This indicates a defect in *pob3(Q308K)* function, and not simply the absence of FACT due to protein instability. The effect of the *pob3(Q308K)* FACT mutation on H3 ChIP is similar to that observed previously with *pob3(L78R)* (10), with the robust nucleosome eviction at 30 min in wild-type at −1297 and −1207 only modestly diminished, and the eviction in URS2 severely reduced (Figure 6A). Nucleosome eviction at other time points from this experiment are shown in Supplementary Figure S5, and another replicate of this experiment, with fewer PCR primer sets, is shown in Supplementary Figure S6.

The *HO* activation program initiates in M phase when Swi5 enters the nucleus and binds to these NDRs, recruiting SWI/SNF which evicts nucleosomes in a bidirectional manner. The nearby nucleosomes to the left and right of the Swi5-binding sites, such as −1897 and −1207, are evicted rapidly, with nucleosome depletion clearly visible at the 15 min time point (Figure 6B); the *pob3(Q308K)* mutation has only modest effect on eviction of these nucleosomes. In contrast, more distal nucleosomes, such as at −2408 and −338, are evicted later (Figure 6B); in addition, eviction of the −2408 and −338 nucleosomes is largely eliminated in the *pob3(Q308K)* mutant (Figure 6B). The time courses of nucleosome eviction shown in Supplementary Figure S7 show that nucleosomes closer to the Mesa nucleosomes at −1628 and −1478 are evicted more quickly than more distant nucleosomes, and that eviction of the more distant nucleosomes is strongly FACT-dependent. These results are consistent with our previous results, which showed that eviction of nucleosomes in URS1 was dependent upon SWI/SNF, but independent of FACT and FACT was required only for eviction of more distal nucleosomes (10).

### FACT is recruited to two promoter regions

**Figure 7.**
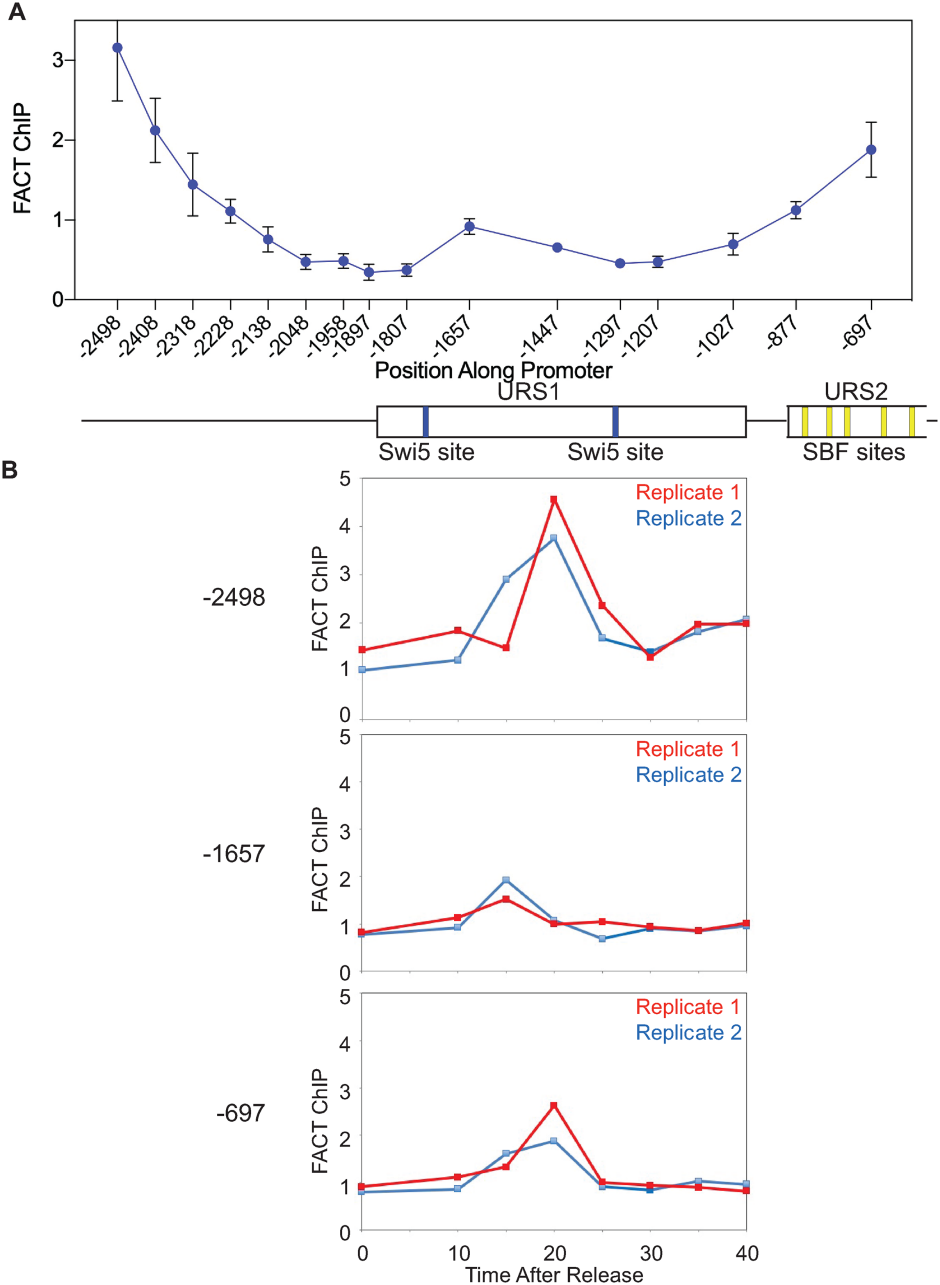
FACT is recruited to multiple regions of the *HO* promoter. (**A**) Cells with an Spt16-Myc tag were synchronized with a *GALp:CDC20* arrest and release, and samples harvested at 20 min after release were analyzed for FACT binding by ChIP. PCR primers were used that span the *HO* promoter, with a promoter diagram below. The error bars reflect the standard deviation of three biological samples. (**B**) FACT recruitment to three promoter positions, centered at −2498, −1657 and −697, was measured as a function of cell cycle time after release from a G2/M arrest. The results are shown for two replicate cultures, which are the same as analyzed for the 20 min time point in part A; there was insufficient DNA for the samples from the third replicate to be assayed.

FACT is recruited to URS2 transiently during the cell cycle, at 20 min after release from a G2/M arrest (Figure 3C). Our previous work showed that FACT recruitment to URS2 is dependent on Swi5 binding and recruitment of the SWI/SNF remodeling complex (10). Based upon the observed FACT-dependent bidirectional nucleosome eviction (Figure 6), we examined whether FACT is recruited to regions of the *HO* promoter in addition to URS2. Spt16-Myc-tagged cells were synchronized, and samples collected for ChIP at 20 min after release from G2/M arrest. FACT binding was analyzed with PCR primers along the *HO* promoter from −2500 to −700. In addition to the FACT present at URS2, there was very strong FACT occupancy at −2498 and a weak binding event at −1650 in the vicinity of the stable nucleosomes (Figure 7A). Both Figure 3C and our previous work (10) showed that FACT recruitment to *HO* is transient during the cell cycle. We tested whether FACT association at −2500 and −1650 was similarly restricted within the cell cycle. Figure 7B shows time course analysis for two replicates. Peak FACT recruitment to both −2500 and −700 occurs at 20 min after release (Figure 7B). Interestingly, it appears that peak FACT recruitment at −1650 occurs slightly earlier, at 15 min after release. Importantly, nucleosome eviction at both −2500 and −700 is FACT-dependent, and FACT occupies these regions at the time then this nucleosome eviction is just beginning. Cumulatively, these results show that FACT is recruited to regions of the *HO* promoter where FACT-dependent nucleosome eviction occurs.

### Dynamic loop formation at the *HO* promoter

**Figure 8.**
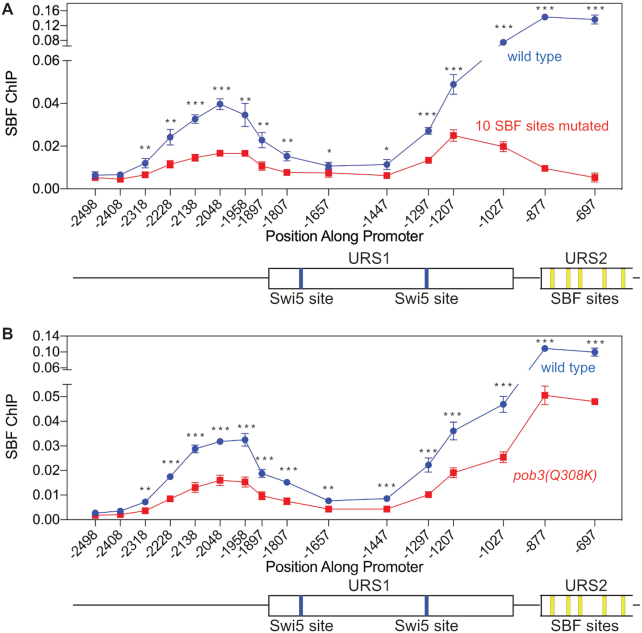
Detection of SBF binding to a promoter region lacking SBF binding sites suggests a promoter loop. (**A**) Mutation of SBF binding sites at URS2 sharply reduces SBF binding at −2050. Two strains with a Swi4-V5 tag were used to measure SBF binding to the *HO* promoter by ChIP assays. The blue symbols show SBF binding in the wild-type strain, and the red symbols show SBF binding in a strain with mutations in 10 SBF binding sites, the nine SBF sites in URS2 and the SBF site at −1166 at the right end of URS1 (23). The error bars reflect the standard deviation of four biological samples. *P < 0.05, **P < 0.01, ***P < 0.001. (**B**) A *pob3(Q308K)* mutation reduces SBF binding at −2050. Two strains with a Swi4-V5 tag were used to measure SBF binding to the *HO* promoter by ChIP assays. The blue symbols show SBF binding in the wild-type strain, and the red symbols show SBF binding in the *pob3(Q308K)* mutant. The error bars reflect the standard deviation of four biological samples. **P < 0.01, ***P < 0.001.

What could lead to the observed bidirectional nucleosome eviction at *HO*? One possibility is that the promoter forms a loop, with the two stable nucleosomes at −1628 and −1478 possibly acting as a pivot point or to organize the arms of the loop. To address this question, we performed SBF ChIP experiments using cells with a Swi4-V5 tag, assuming that a promoter loop would place SBF bound at URS2 in proximity to DNA sequences upstream of URS1 and thus allow a ChIP signal at this upstream region even though it lacks discernible SBF binding sites in this upstream region, using the well-characterized consensus SBF binding site (54–56). As shown in Figure 8A (blue), in addition to the strong SBF ChIP binding seen at URS2, there is also significant SBF binding between −2138 and −1958 upstream of URS1. To determine whether SBF bound at URS2 is driving this upstream ChIP signal, we repeated this experiment using a strain with mutations in all of the SBF sites. This experiment shows that eliminating the URS2 SBF sites results in a dramatic loss of SBF binding at the upstream −2050 region (Figure 8A, red).

We next asked whether the *pob3(Q308K)* FACT mutation would affect SBF binding in the −2050 region. SBF binding on the *HO* promoter was measured by ChIP, comparing wild-type and *pob3(Q308K)* (Figure 8B). The results show that FACT is required for efficient SBF binding to both URS2 as well as the upstream −2050 region. While other explanations are possible, these results are consistent with a physical interaction between the upstream and downstream promoter regions, as expected for a looped structure.

### Possible role for FACT in promoting a loop

The yeast *HO* gene was instrumental in establishing the principle of sequential eviction of nucleosomes along a promoter during the cell cycle (10). Here we show that this nucleosome eviction at the *HO* promoter is bidirectional, originating from two stable nucleosomes centered at −1628 and −1478, and extending for nearly 1000 bp in each direction. Nucleosomes closer to the initiating site are evicted sooner during the cell cycle compared to those nucleosomes that are further away, suggesting sequential progression of eviction in parallel on both arms of a loop. Importantly, eviction of the closer nucleosomes is FACT-independent, while eviction of the more distant nucleosomes requires the FACT histone chaperone. We have previously shown that FACT is recruited to URS2 transiently during the cell cycle, and here we show that FACT is also recruited much further upstream, near −2500. The variable dependence on FACT suggests distinct mechanisms for evictions at different promoter regions.

What is the significance of this FACT-dependent bidirectional nucleosome eviction? One possibility is that the stable nucleosomes centered at −1550 function as an initiation site in forming a DNA loop. Evidence supporting loop formation includes the experiments in Figure 8 suggesting that the upstream region near −2050 is in sufficiently close proximity to the SBF sites in URS2 for a SBF ChIP signal to be detected at the upstream region. Importantly, mutation of all of the SBF sites in URS2 nearly eliminates SBF binding at the upstream −2050 region.

The pattern and timing of FACT recruitment to the *HO* promoter during the cell cycle is notable. FACT is not detected at the promoter at the G2/M arrest. At 20 min following release, there is robust FACT binding to the −2500 and −700 regions of the promoter. Interestingly, at 15 min there is weak binding to the stable nucleosome region at −1550; it is not clear whether this binding is requisite for the subsequent binding at −2500 and −700. Significantly, the FACT binding at −2500 and −700 occur at a time when nucleosome eviction in these regions is just beginning, and the *pob3(Q308K)* FACT mutation largely eliminates eviction of nucleosomes in these regions more distant from the −1550 initiation site region. This result suggests that FACT is required for formation of a promoter loop. Further work is needed to understand this role of FACT in modulating promoter structure.

## Supplementary Material

gkaa819_Supplemental_File

## Notes

Present address: Robert M. Yarrington, New England Biolabs, 240 County Road, Ipswich, MA 01938-2723, USA.
