# Supplementary material for: FACT and Ash1 promote long-range and bidirectional nucleosome eviction at the HO promoter
Source: Nucleic Acids Res. 2020 Oct 3;48(19):10877–89. doi: 10.1093/nar/gkaa819 (PMC7641740; doi:10.1093/nar/gkaa819)
Supplement: gkaa819_Supplemental_File [file gkaa819_supplemental_file.pdf]

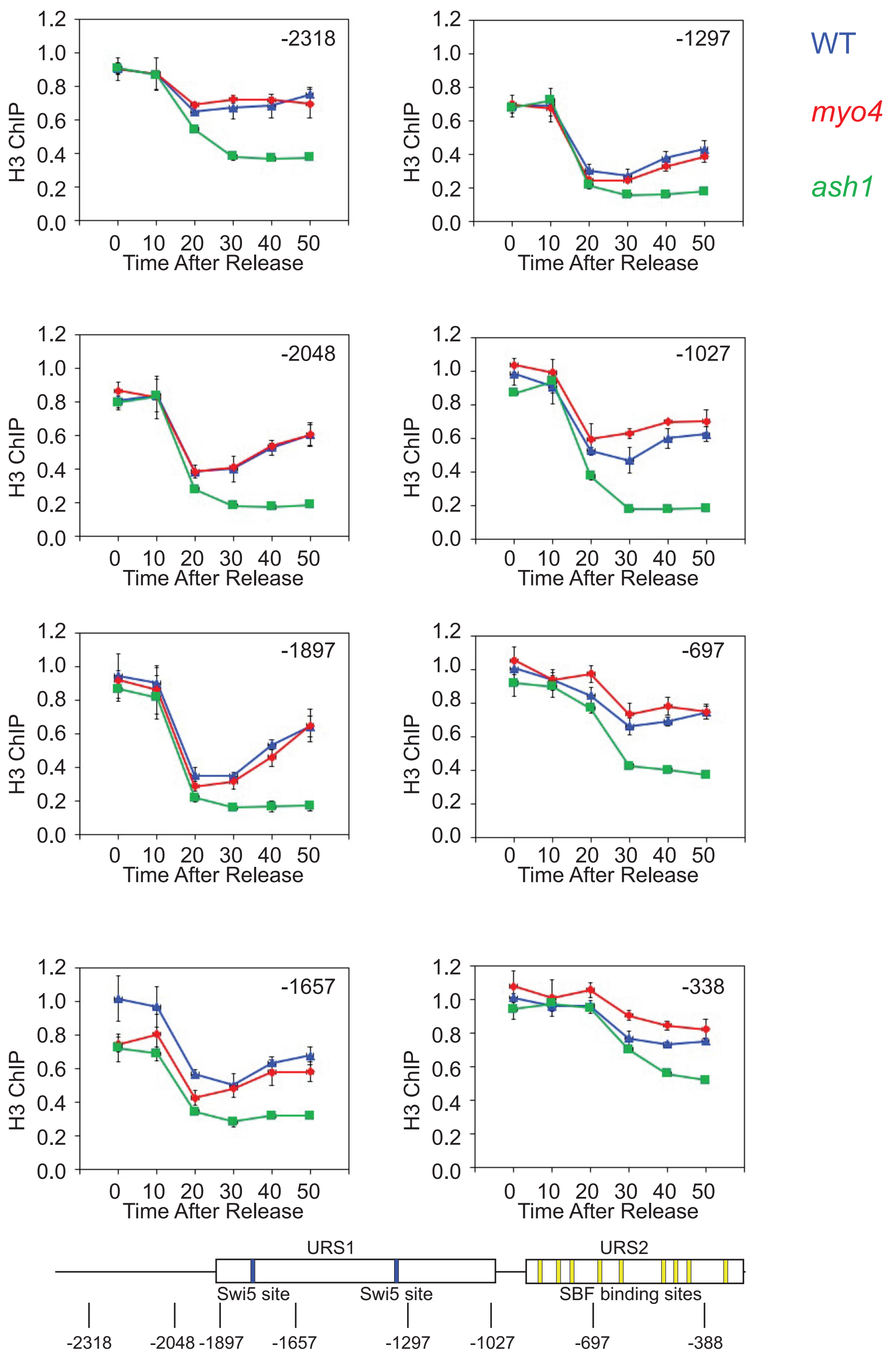
WT
myo4
ash1
-2318
-1297
-2048
-1027
-1897
-697
-1657
-338
H3 ChIP
Time After Release
URS1
URS2
Swi5 site
Swi5 site
SBF binding sites
-2318
-2048 -1897
-1657
-1297
-1027
-697
-388

Supplemental Fig S1. Nucleosome eviction to the left of URS1 is more pronounced in an *ash1* mutant.

This figure contains the data from Figure 4 replotted as a function of time on the x-axis. Wild type, myo4, and ash1 cells were synchronized with a *GALp:CDC20* arrest and release, and samples harvested at the indicated timepoints were used for H3 ChIP to determine nucleosome occupancy. PCR primers were used that span the *HO* promoter, with a promoter diagram at the bottom. Wild type blue, *myo4* red, *ash1* green. ChIP values were first normalized to their input DNA sample and then to the ChIP signal at the IGR-I negative control region.

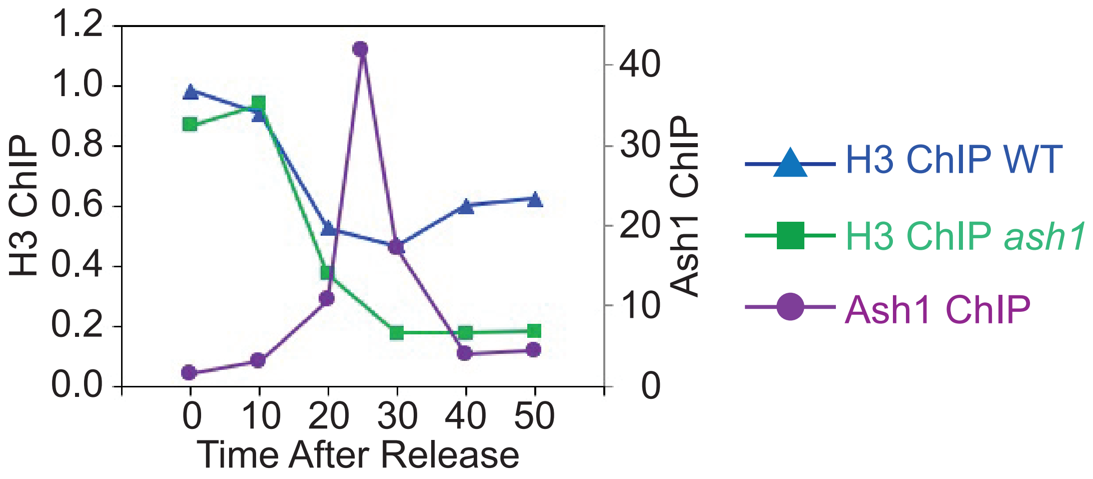

Supplemental Fig S2. The effect of the *ash1* mutation on nucleosome structure persists after the Ash1 protein is largely gone from the cell.

This figure contains the Ash1 ChIP data from Figure 2B combined with the H3 ChIP data from Figure 4B for wild type at promoter position -1027.

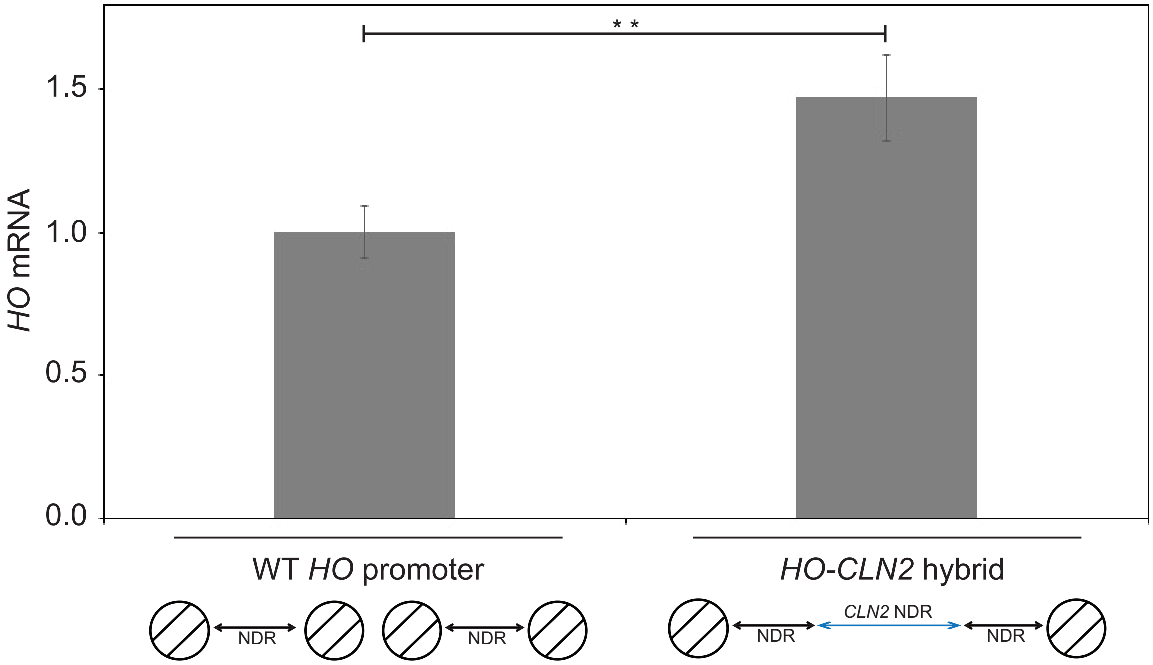

Supplemental Fig S3. Expression from the *HO-CLN2* promoter lacking nucleosomes between the two Swi5 binding sites is increased relative to wild type.

HO mRNA levels were measured by RT-qPCR from strains with either the wild type *HO* promoter or the *HO-CLN2* hybrid promoter lacking the two Mesa nucleosomes. The error bars reflect the standard deviation of three biological samples. ** $p < 0.01$.

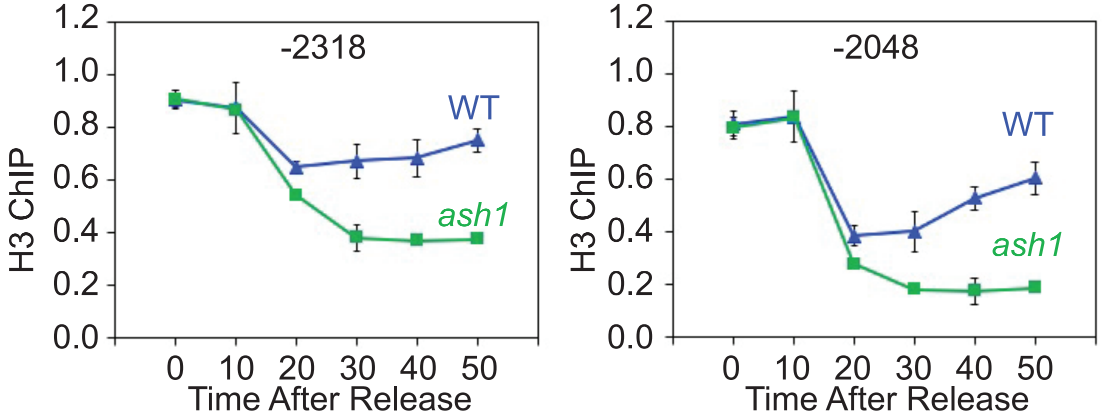

Supplemental Fig S4. Nucleosome eviction to the left of URS1 is more pronounced in an *ash1* mutant.

Wild type and *ash1* cells were synchronized with a *GALp:CDC20* arrest and release, and samples harvested at the indicated timepoints were used for H3 ChIP to determine nucleosome occupancy. The wild type and *ash1* H3 ChIP data from Fig 4A for the -2318 and -2048 promoter positions are replotted as a function of time after release from the arrest. Wild type blue, *ash1* green.

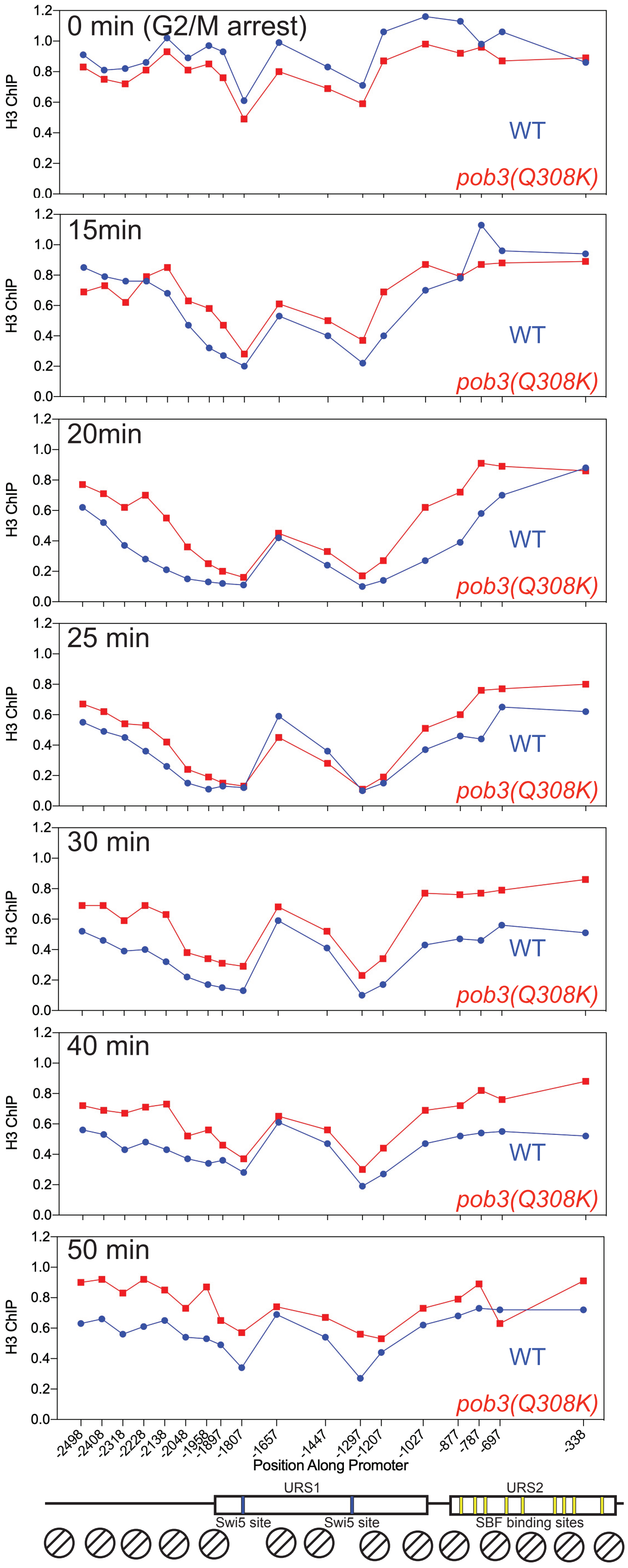
0 min (G2/M arrest)
15min
20min
25 min
30 min
40 min
50 min
H3 ChIP
WT
pob3(Q308K)
1.2
1.0
0.8
0.6
0.4
0.2
0.0
-2498 -2408 -2318 -2228 -2138 -2048 -1958 -1897 -1807 -1657 -1447 -1297 -1207 -1027 -877 -787 -697 -338
Position Along Promoter
URS1
URS2
Swi5 site
Swi5 site
SBF binding sites

Supplemental Fig S5. A *pob3-Q308K* mutation reduces nucleosome eviction in both directions.

Wild type and *pob3-Q308K* cells were synchronized with a *GALp:CDC20* arrest and release, and samples harvested at the indicated timepoints were used for H3 ChIP to determine nucleosome occupancy. PCR primers were used that span the *HO* promoter, with a promoter diagram at the bottom. Wild type blue, *pob3-Q308K* red. The data for the 0 min and 30 min timepoints are also shown in Fig 6A.

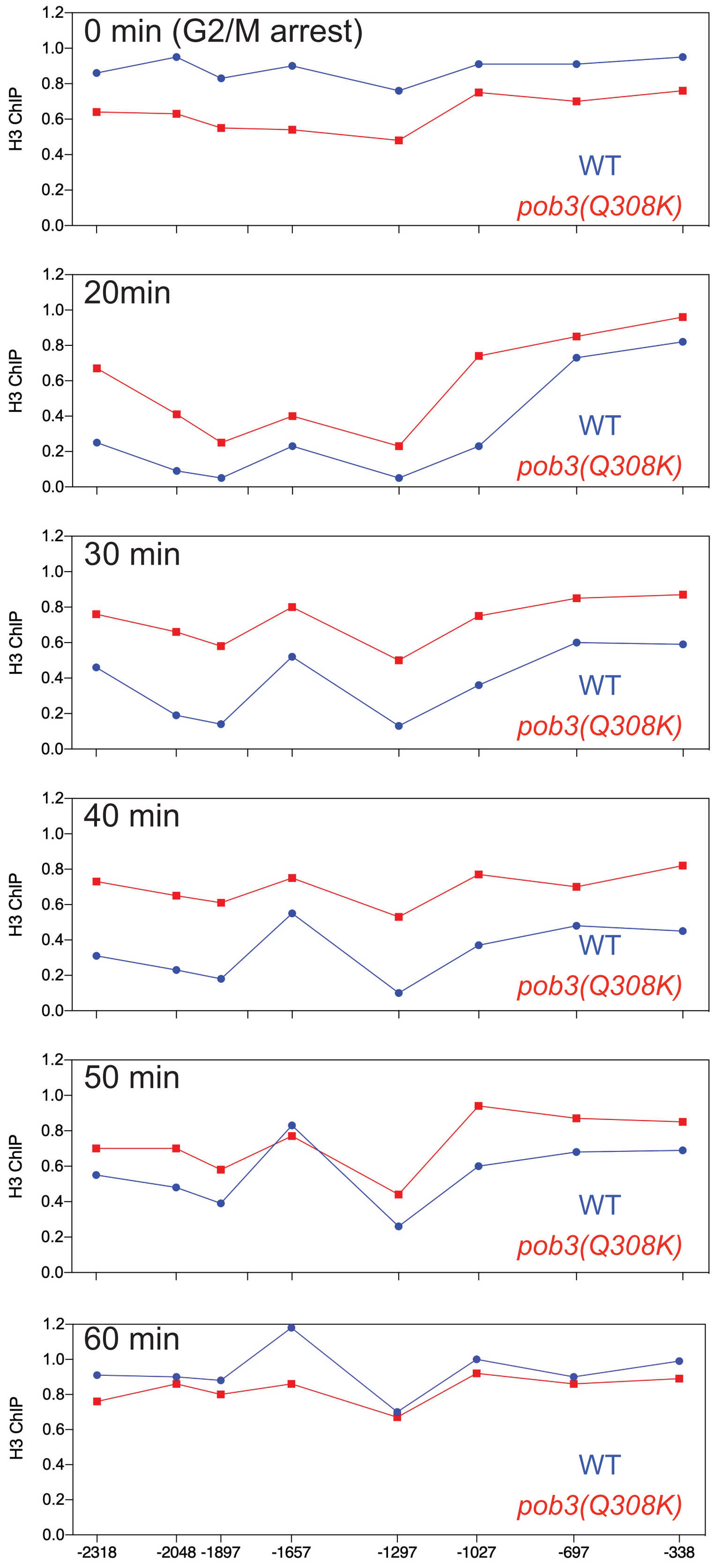
0 min (G2/M arrest)
20min
30 min
40 min
50 min
60 min
H3 ChIP
WT
pob3(Q308K)
1.2
1.0
0.8
0.6
0.4
0.2
0.0
-2318
-2048 -1897
-1657
-1297
-1027
-697
-338

Supplemental Fig S6. A *pob3-Q308K* mutation reduces nucleosome eviction in both directions.

Wild type and *pob3-Q308K* cells were synchronized with a *GALp:CDC20* arrest and release, and samples harvested at various timepoints were used for H3 ChIP to determine nucleosome occupancy. PCR primers were used that span the *HO* promoter. Wild type blue, *pob3-Q308K* red. ChIP values were first normalized to their input DNA sample and then to the ChIP signal at the IGR-I negative control region. This is a replica of the experiment in Fig 6A, but with fewer PCR primer sets.

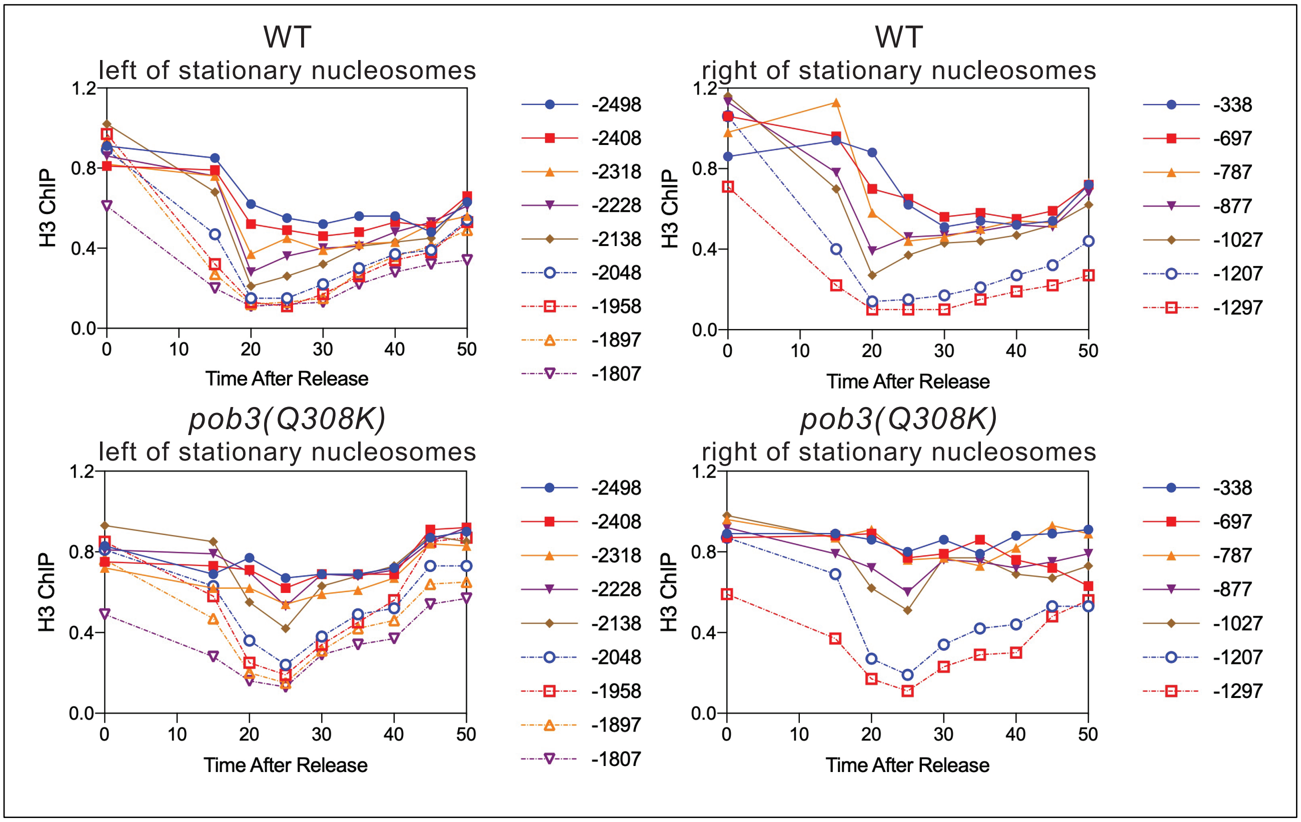

Supplemental Fig S7. Nucleosomes closer to the NDRs are evicted more quickly, and eviction of distant nucleosomes is highly FACT-dependent.

Wild type and *pob3-Q308K* cells were synchronized with a *GALp:CDC20* arrest and release, and samples harvested at various timepoints were used for H3 ChIP to determine nucleosome occupancy. The centers of the PCR amplicons along the *HO* promoter are indicated. This the same data shown in Fig 6A, but replotted as a function of time after release from the arrest. The left panels show nucleosomes to the left of the stationary nucleosomes at -1628 and -1478, and the right panels show nucleosomes to the right of the stationary nucleosomes. The upper panels show data from wild type, and the lower panels show *pob3-Q308K*.

Supplemental Table S1. Strains used in this study.

Figure 1A	
DY18407	*MAT***a** *ASH1-V5::HIS3MX ade2 can1 his3 leu2 trp1 ura3*
DY18520	*MAT***a** *ASH1-V5::HIS3MX myo4::URA3 ade2 can1 his3 leu2 trp1 ura3*
Figure 1B	
DY6546	*MAT***a** *GALp::CDC20::ADE2 SWI5-Myc::HIS3MX ade2 can1 his3 leu2 trp1 ura3*
DY8032	*MAT*α *GALp::CDC20::ADE2 SWI5-Myc::HIS3MX myo4::URA3 ade2 can1 his3 leu2 trp1 ura3*
Figure 2A	
DY18407	*MAT***a** *ASH1-V5::HIS3MX ade2 can1 his3 leu2 trp1 ura3*
DY18520	*MAT***a** *ASH1-V5::HIS3MX myo4::URA3 ade2 can1 his3 leu2 trp1 ura3*
Figure 2B	
DY18522	*MAT***a** *GALp::CDC20::ADE2 ASH1-V5::HIS3MX ade2 can1 his3 leu2 trp1 ura3*
DY17939	*MAT***a** *GALp::CDC20::ADE2 ASH1-V5::HIS3MX myo4::URA3 ade2 can1 his3 leu2 trp1 ura3*
Figure 2C	
DY18420	*MAT***a** *SWI4-V5::HIS3MX ade2 can1 his3 leu2 trp1 ura3*
DY18454	*MAT***a** *SWI4-V5::HIS3MX myo4::URA3 ade2 can1 his3 leu2 trp1 ura3*
Figure 2D	
DY6546	*MAT***a** *GALp::CDC20::ADE2 SWI5-Myc::HIS3MX ade2 can1 his3 leu2 trp1 ura3*
DY8032	*MAT*α *GALp::CDC20::ADE2 SWI5-Myc::HIS3MX myo4::URA3 ade2 can1 his3 leu2 trp1 ura3*

Figure 3A

DY18327	*MATα SWI2-V5::HphMX ade2 can1 his3 leu2 lys2 met15 trp1 ura3*
DY18328	*MAT***a** *SWI2-V5::HphMX myo4::URA3 ade2 can1 his3 leu2 met15 trp1 ura3*

Figure 3B

DY18334	*MAT***a** *GCN5-V5::HphMX ade2 can1 his3 leu2 met15 trp1 ura3*
DY18336	*MAT***a** *GCN5-V5::HphMX myo4::URA3 ade2 can1 his3 leu2 lys2 trp1 ura3*

Figure 3C

DY14124	*MAT***a** *GALp::CDC20::ADE2 SPT16-MYC::HIS3MX ade2 can1 his3 leu2 trp1 ura3*
DY19595	*MAT***a** *GALp::CDC20::ADE2 SPT16-MYC::HIS3MX myo4::URA3 ade2 can1 his3 leu2 trp1 ura3*

Figure 4

DY18360	*MAT***a** *GALp::CDC20::ADE2 GCN5-V5::HIS3MX ade2 can1 his3 leu2 lys2 met15 trp1 ura3*
DY18364	*MAT***a** *GALp::CDC20::ADE2 GCN5-V5::HIS3MX myo4::URA3 ade2 can1 his3 leu2 lys2 met15 trp1 ura3*
DY18362	*MAT***a** *GALp::CDC20::ADE2 GCN5-V5::HIS3MX ash1::LEU2 ade2 can1 his3 leu2 lys2 met15 trp1 ura3*

Figure 5A

DY6669	*MAT***a** *GALp::CDC20::ADE2 ade2 can1 his3 leu2 trp1 ura3*

Figure 5B

DY150	*MAT***a** *ade2 can1 his3 leu2 trp1 ura3*

DY3923 *MAT***a** *ace2::HIS3 ade2 can1 his3 leu2 lys2 trp1 ura3*

DY10318 *MAT***a** *swi5::TRP1 ade2 can1 his3 leu2 trp1 ura3*

DY14529 *MAT***a** *ace2::HIS3 swi5::TRP1 ade2 can1 his3 leu2 trp1 ura3*

DY19583 *MAT***a** *HO[-1725 to -1398 deleted]:CLN2[-764 to -435, mutSBF-wtNDR]:: KanMX(3') ade2 can1 his3 leu2 trp1 ura3*

DY19588 *MAT***a** *HO[-1725 to -1398 deleted]:CLN2[-764 to -435, mutSBF-wtNDR]:: KanMX(3') ace2::HIS3 ade2 can1 his3 leu2 trp1 ura3*

DY19589 *MAT***a** *HO[-1725 to -1398 deleted]:CLN2[-764 to -435, mutSBF-wtNDR]:: KanMX(3') swi5::TRP1 ade2 can1 his3 leu2 trp1 ura3*

DY19591 *MAT***a** *HO[-1725 to -1398 deleted]:CLN2[-764 to -435, mutSBF-wtNDR]:: KanMX(3') ace2::HIS3 swi5::TRP1 ade2 can1 his3 leu2 trp1 ura3*

Figure 6

DY17461 *MAT***a** *GALp::CDC20::ADE2 SWI4-V5::HIS3MX ade2 can1 his3 leu2 trp1 ura3*

DY17962 *MAT***a** *GALp::CDC20::ADE2 SWI4-V5::HIS3MX pob3(Q308K)::KanMX ade2 can1 his3 leu2 lys2 trp1 ura3*

Figure 7

DY14124 *MAT***a** *GALp::CDC20::ADE2 SPT16-Myc::KanMX ade2 can1 his3 leu2 trp1 ura3*

Figure 8A

DY19614 *MAT***a** *SWI4-V5::HIS3MX HO::KanMX(3') ade2 can1 his3 leu2 trp1 ura3*

DY19748 *MAT***a** *SWI4-V5::HIS3MX HO(10XSBFmut)::KanMX(3') ade2 can1 his3 leu2 trp1 ura3*

Figure 8B

DY16942 *MAT***a** *SWI4-V5::HIS3MX ade2 can1 his3 leu2 trp1 ura3*

DY17964 *MAT***a** *SWI4-V5::HIS3MX pob3(Q308K)::KanMX ade2 can1 his3 leu2 lys2 trp1 ura3*

Suppl Fig S1

DY18360	*MAT***a** *GALp::CDC20::ADE2 GCN5-V5::HIS3MX ade2 can1 his3 leu2 lys2 met15 trp1 ura3*
DY18364	*MAT***a** *GALp::CDC20::ADE2 GCN5-V5::HIS3MX myo4::URA3 ade2 can1 his3 leu2 lys2 met15 trp1 ura3*
DY18362	*MAT***a** *GALp::CDC20::ADE2 GCN5-V5::HIS3MX ash1::LEU2 ade2 can1 his3 leu2 lys2 met15 trp1 ura3*

Suppl Fig S2

DY18522	*MAT***a** *GALp::CDC20::ADE2 ASH1-V5::HIS3MX ade2 can1 his3 leu2 trp1 ura3*
DY18360	*MAT***a** *GALp::CDC20::ADE2 GCN5-V5::HIS3MX ade2 can1 his3 leu2 lys2 met15 trp1 ura3*
DY18362	*MAT***a** *GALp::CDC20::ADE2 GCN5-V5::HIS3MX ash1::LEU2 ade2 can1 his3 leu2 lys2 met15 trp1 ura3*

Suppl Fig S3

DY150	*MAT***a** *ade2 can1 his3 leu2 trp1 ura3*
DY19583	*MAT***a** *HO[-1725 to -1398 deleted]:CLN2[-764 to -435, mutSBF-wtNDR]:: KanMX(3')* *ade2 can1 his3 leu2 trp1 ura3*

Suppl Fig S4

DY18360	*MAT***a** *GALp::CDC20::ADE2 GCN5-V5::HIS3MX ade2 can1 his3 leu2 lys2 met15 trp1 ura3*
DY18362	*MAT***a** *GALp::CDC20::ADE2 GCN5-V5::HIS3MX ash1::LEU2 ade2 can1 his3 leu2 lys2 met15 trp1 ura3*

Suppl Fig S5

DY17461 *MAT***a** *GALp::CDC20::ADE2 SWI4-V5::HIS3MX ade2 can1 his3 leu2 trp1 ura3*

DY17962 *MAT***a** *GALp::CDC20::ADE2 SWI4-V5::HIS3MX pob3(Q308K)::KanMX ade2 can1 his3 leu2 lys2 trp1 ura3*

Suppl Fig S6

DY17461 *MAT***a** *GALp::CDC20::ADE2 SWI4-V5::HIS3MX ade2 can1 his3 leu2 trp1 ura3*

DY17962 *MAT***a** *GALp::CDC20::ADE2 SWI4-V5::HIS3MX pob3(Q308K)::KanMX ade2 can1 his3 leu2 lys2 trp1 ura3*

Suppl Fig S7

DY17461 *MAT***a** *GALp::CDC20::ADE2 SWI4-V5::HIS3MX ade2 can1 his3 leu2 trp1 ura3*

DY17962 *MAT***a** *GALp::CDC20::ADE2 SWI4-V5::HIS3MX pob3(Q308K)::KanMX ade2 can1 his3 leu2 lys2 trp1 ura3*

Supplemental Table S2. Oligonucleotides used in this study.

RT-qPCR Oligos

F1066	*HO* ORF (s)	AAATGGAGCGCTCTAAAGGAGAA
F1067	*HO* ORF (as)	CTAACCACAGACCAAGCATCCAA
F2430	RPR1 control (s)	CACCTATGGGCGGGTTATCAG
F2431	RPR1 control (as)	CCTAGGCCGAACTCCGTGA

ChIP-qPCR Oligos

F3307	*HO* Promoter -2546 to -2523 (s)	CTATAAGGTTGATATTCTCACGAG
F3312	*HO* Promoter -2479 to -2448 (as)	TAGCAATTATAATCTCTAATATACTATTTCTG
F3313	*HO* Promoter -2456 to -2425 (s)	TAATTGCTAATCAAATCAAAATATAAAATTAG
F3314	*HO* Promoter -2382 to -2358 (as)	GGTTTGCAGAAATATCATTAAAAAA
F3315	*HO* Promoter -2366 to -2345 (s)	CTGCAAACCAAAGAAAGATTGT
F3316	*HO* Promoter -2290 to -2268 (as)	TTTGAATTATGAGGCCCAGATAT
F3317	*HO* Promoter -2276 to -2256 (s)	TAATTCAAAAGACGGTGCCAT
F3318	*HO* Promoter -2200 to -2178 (as)	GTTGCAATGAGACCATTCTTTTC
F3319	*HO* Promoter -2186 to -2165 (s)	CATTGCAACACGTAAGGTTAAG
F3320	*HO* Promoter -2100 to -2088 (as)	CTCGTTCTCCCTACTATTTCACC
F3321	*HO* Promoter -2096 to -2071 (s)	GAGAACGAGTACCTGTAGTAAAAAGT
F3322	*HO* Promoter -2018 to -1998 (as)	ATGTTGGCGTTTGTCTCGAAC
F3323	*HO* Promoter -2006 to -1984 (s)	CGCCAACATTTTTGTTTCTTTTG
F3324	*HO* Promoter -1929 to -1908 (as)	CTTTGCCAGTAAGAACTACGTG
F1901	*HO* Promoter -1946 to -1927 (s)	GATCTAATGTAGAGTTGCAC
F1921	*HO* Promoter -1866 to -1847 (as)	GTTGGAACTAAACGGTAAAG
F3114	*HO* Promoter -1856 to -1834 (s)	TAGTTCCAACGTAAAATTGTGCC
F1924	*HO* Promoter -1776 to -1757 (as)	TAACGCGGCAAAACATATTA
F1909	*HO* Promoter -1707 to -1688 (s)	GAATAACGAACATTCATAGC

F1929	*HO* Promoter -1627 to -1608 (as)	TATGACTAACATAGAGAGTA
F1916	*HO* Promoter -1497 to -1478 (s)	AGAGCTCATAATTCAAGCAA
F1936	*HO* Promoter -1417 to -1398 (as)	AACATAATTCCAGCACGCAG
F1998	*HO* Promoter -1347 to -1328 (s)	GCCGGTGCCTGCGATGAGAT
F2002	*HO* Promoter -1267 to -1248 (as)	ACTGTTGAGGTCTTTTCTAT
F2093	*HO* Promoter -1295 to -1270 (s)	AATGCTGGAGCAAAAATTTCAATCAG
F2094	*HO* Promoter -1142 to -1122 (as)	GGAGCCCCTCAGACATTAGCC
F2001	*HO* Promoter -1257 to -1238 (s)	CCTCAACAGTAATTAACCCA
F2005	*HO* Promoter -1177 to -1158 (as)	TTTTACGCGATTCGGCCCAA
F1954	*HO* Promoter -1077 to -1058 (s)	AGTAATATTCCCAAGAAAGA
F1977	*HO* Promoter -997 to -978 (as)	GATCGAACTTACTCAATAGC
F1959	*HO* Promoter -927 to -908 (s)	GAGGTTGGTATTGATTGTTG
F1982	*HO* Promoter -847 to -828 (as)	GTCAGGGTATGAACCATACG
F1962	*HO* Promoter -837 to -818 (s)	ATACCCTGACTTGGCAAACC
F1963	*HO* Promoter -757 to -738 (as)	GTCGTCGATTAGCGGATCAC
F2099	*HO* Promoter -839 to -819 (s)	TCATACCCTGACTTGGCAAAC
F2100	*HO* Promoter -643 to -620 (as)	CTTAAGCCCTGTGTAGGATTGATT
F1965	*HO* Promoter -747 to -728 (s)	ATGTGAATGAATACATGAAA
F1988	*HO* Promoter -667 to -648 (as)	TTTTCGTGACGCACATGTCT
F2105	*HO* Promoter -448 to -423 (s)	GGTTTACGAAATGATCCACGAAAATC
F2106	*HO* Promoter -273 to -251 (as)	TTTCACACCTAATAACGCCCAGC
F1855	*HO* Promoter -387 to -368 (s)	ATGTCATGTCCACATTAACA
F1876	*HO* Promoter -307 to -288 (as)	AAGTAGTAGGTACCAATGGT
F1070	Control *ACT1* (s)	CTGCCGGTATTGACCAAACTACTTACAA
F1071	Control *ACT1* (as)	GTCAAAGAAGCCAAGATAGAACCACCAA
F1399	Control IG-1 (s)	GGCTGTCAGAATATGGGGCCGTAGTA
F1400	Control IG-1 (as)	CACCCCGAAGCTGCTTTCACAATAC

<u>MNase qPCR Oligos</u>

F1901	*HO* Promoter -1947 to -1928 (s)	GATCTAATGTAGAGTTGCAC
F1921	*HO* Promoter -1867 to -1848 (as)	GTTGGAACTAAACGGTAAAG
F1902	*HO* Promoter -1917 to -1898 (s)	CTGGCAAAGAAATCGATGCA
F1922	*HO* Promoter -1837 to -1818 (as)	GCCATTTTAAGTCCAAAGGC
F1903	*HO* Promoter -1887 to -1868 (s)	GAATAAAGGTGATATTTGAT
F1923	*HO* Promoter -1807 to -1788 (as)	TTTGGAAAAATAAAGAGTTA
F1904	*HO* Promoter -1857 to -1838 (s)	TAGTTCCAACGTAAAATTGT
F1924	*HO* Promoter -1777 to -1758 (as)	TAACGCGGCAAAACATATTA
F1905	*HO* Promoter -1827 to -1808 (s)	TTAAAATGGCGTGGCAGAAC
F1925	*HO* Promoter -1747 to -1728 (as)	TCTTGATCCGCCTTTTTTTG
F1906	*HO* Promoter -1797 to -1778 (s)	TTTTTCCAAATCAGAAAAAT
F1926	*HO* Promoter -1717 to -1698 (as)	TTCGTTATTCTACGCACTTT
F1907	*HO* Promoter -1767 to -1748 (s)	TGCCGCGTTAAAACCTACAT
F1927	*HO* Promoter -1687 to -1668 (as)	TTTGTAAAGCCTCCAGAACA
F1908	*HO* Promoter -1737 to -1718 (s)	CGGATCAAGATGTATGAAAG
F1928	*HO* Promoter -1657 to -1638 (as)	GGATAAGATCGCACCTAACA
F1909	*HO* Promoter -1707 to -1688 (s)	GAATAACGAACATTCATAGC
F1929	*HO* Promoter -1627 to -1608 (as)	TATGACTAACATAGAGAGTA
F1910	*HO* Promoter -1677 to -1658 (s)	GCTTTACAAAAGGTAATCTT
F1930	*HO* Promoter -1597 to -1578 (as)	GAAAATAATTCTCTCACAGA
F1911	*HO* Promoter -1647 to -1628 (s)	GATCTTATCCGAAAAGCAAT
F1931	*HO* Promoter -1567 to -1548 (as)	TAAAGGAACCATGTGATCTT
F1912	*HO* Promoter -1617 to -1598 (s)	GTTAGTCATACAAACTGACT
F1932	*HO* Promoter -1537 to -1518 (as)	GTCATATAATGGAATGATAG
F1913	*HO* Promoter -1587 to -1568 (s)	AATTATTTTCATATCAACGT
F1933	*HO* Promoter -1507 to -1488 (as)	TATGAGCTCTAAGATTCAAG
F1914	*HO* Promoter -1557 to -1538 (s)	GGTTCCTTTATCAAGTACTA

F1934	*HO* Promoter -1477 to -1458 (as)	GAAATTCTTAGCTTCGCAAC
F1915	*HO* Promoter -1527 to -1508 (s)	ATTATATGACCTATTTACTT
F1935	*HO* Promoter -1447 to -1428 (as)	TAATGAAGATTGTTAAGTTC
F1916	*HO* Promoter -1497 to -1478 (s)	AGAGCTCATAATTCAAGCAA
F1936	*HO* Promoter -1417 to -1398 (as)	AACATAATTCCAGCACGCAG
F1917	*HO* Promoter -1467 to -1448 (s)	TAAGAATTTCACATGTTGTT
F1937	*HO* Promoter -1387 to -1368 (as)	GGGAAAAATGAAAAAAAGGA
F1918	*HO* Promoter -1437 to -1418 (s)	ATCTTCATTATACCCAATCG
F1938	*HO* Promoter -1357 to -1338 (as)	AGGCACCGGCAGTACAGTGC
F1919	*HO* Promoter -1407 to -1388 (s)	GAATTATGTTAAAAGTTACA
F1939	*HO* Promoter -1327 to -1308 (as)	TTTTTTTTTTAAATTGATGT
F1920	*HO* Promoter -1377 to -1358 (s)	CATTTTTCCCTACGCTCAGG
F1940	*HO* Promoter -1297 to -1278 (as)	AATTTTTGCTCCAGCATTAT
F1998	*HO* Promoter -1347 to -1328 (s)	GCCGGTGCCTGCGATGAGAT
F2002	*HO* Promoter -1267 to -1248 (as)	ACTGTTGAGGTCTTTTCTAT
F1999	*HO* Promoter -1317 to -1298 (s)	AAAAAAAAAACCAGCATGCT
F2003	*HO* Promoter -1237 to -1218 (as)	TCGATTATTTGATACCCCTT
F2000	*HO* Promoter -1287 to -1268 (s)	AGCAAAAATTTCAATCAGAA
F2004	*HO* Promoter -1207 to -1188 (as)	CACAGATCATCCGTAGAGTG
F2001	*HO* Promoter -1257 to -1238 (s)	CCTCAACAGTAATTAACCCA
F2005	*HO* Promoter -1177 to -1158 (as)	TTTTACGCGATTCGGCCCAA
F1949	*HO* Promoter -1227 to -1208 (s)	AAATAATCGATGTGCTTTTT
F1972	*HO* Promoter -1147 to -1128 (as)	CCTCAGACATTAGCCGCCAC
F1951	*HO* Promoter -1167 to -1148 (s)	TCGCGTAAAAAGTTTGATTC
F1974	*HO* Promoter -1087 to -1068 (as)	GAATATTACTTGGACATTTT
F1952	*HO* Promoter -1137 to -1118 (s)	ATGTCTGAGGGGCTCCAACA
F1975	*HO* Promoter -1057 to -1038 (as)	TTATCAAAGCACTCTGCGGT
F1953	*HO* Promoter -1107 to -1088 (s)	GCCTCATTTCTTGAGGGCAC

F1976 *HO* Promoter -1027 to -1008 (as) ACAAAACCTACGTTAAGACC

F1954 *HO* Promoter -1077 to -1058 (s) AGTAATATTCCCAAGAAAGA

F1977 *HO* Promoter -997 to -978 (as) GATCGAACTTACTCAATAGC

<u>Epitope Tagging Oligos</u>

F759 *GAL11* Tagging (s)

GAACAATTCAATGTATGGGATTGGAATAATTGGACAAGTGCTACTCGGATCCCCGGGTTAATTAA

F760 *GAL11* Tagging (as)

AAGTAACTTCAAAAGTATCAAAAGTATGGAAACTTCAAATGTTCAGAATTCGAGCTCGTTTAAAC

F732 *GCN5* Tagging (s)

AATAATAAAGTAAAAGAAATACCTGAATATTCTCACCTTATTGATCGGATCCCCGGGTTAATTAA

F733 *GCN5* Tagging (as)

TTTCTTCTTCGAAAGGAATAGTAGCGGAAAAGCTTCTTCTACGCAGAATTCGAGCTCGTTTAAAC

F810 *SRB4* Tagging (s)

CTTCCTACATTTTATTGTCGCTGAGTACATCCAGCAAAAGAAGGTGCGTACGCTGCAGGTCGAC

F811 *SRB4* Tagging (as)

GGCATTCTATGGCAATGTATGTAGGTTTAAGGAGTGACTCAGGATATTAATCGATGAATTCGAGCTCG

F774 *SWI2* Tagging (s)

TCTTTCACAGATGAAGCGGACTCGAGCATGACAGAAGCGAGTGTACGGATCCCCGGGTTAATTAA

F775 *SWI2* Tagging (as)

ATGTTTGTCTACGTATAAACGAATAAGTACTTATATTGCTTTAGGAAGGTAGAATTCGAGCTCGTTTAAAC

F3259 *SWI4* Tagging (s)

GACTCAAAATTGGACGATATAGAAAAGGATTTGAGGGCAAACGCACGGATCCCCGGGTTAATTAA

F3260 *SWI4* Tagging (as)

CTCTGATAATATAGTAAAAATTATTGGTACATTGTGAATTAAAATGAATTCGAGCTCGTTTAAAC

F665 *SWI5* Tagging (s)

AATGGAACGGGGATTATGGTTTCGCCAATGAAAACTAATCAAAGGCGGATCCCCGGGTTAATTAA

F666 *SWI5* Tagging (as)

TGTTACCCACATTCTCCACTCTTCCACAGAAAAAATTCCTAAAGTGAATTCGAGCTCGTTTAAAC

Oligonucleotides are listed in pairs with a sense (s) oligo and an antisense (as) oligo.
